# Pesticide Use and Asthma in Alberta Grain Farmers

**DOI:** 10.3390/ijerph15030526

**Published:** 2018-03-15

**Authors:** Nicola Cherry, Jeremy Beach, Ambikaipakan Senthilselvan, Igor Burstyn

**Affiliations:** 1 Faculty of Medicine, Division of Preventive Medicine, University of Alberta, 5-22 University Terrace, 8303-112 St, Edmonton, AB T6G 1K4, Canada; jeremy.beach@cpsa.ab.ca (J.B.); ib68@drexel.edu (I.B.); 2 School of Public Health, University of Alberta, Edmonton, AB T6G 1C9, Canada; sentil@ualberta.ca; 3 Dornsife School of Public Health, Drexel University, Philadelphia, PA 19104, USA

**Keywords:** pesticides, grain farming, asthma, phenoxy herbicides, administrative health records, likelihood score

## Abstract

A study of the respiratory health of grain farmers in Alberta, Canada was carried out in March 2002. Two populations were identified: members, in 1983, of a province-wide farm organisation, and grain farmers registered with the provincial agriculture department. A telephone interview addressed pesticide use (using pre-circulated trade names), chronic disease and respiratory symptoms. Pesticide ingredients were identified from provincial crop protection guides. Total years of use were calculated for seven chemical groups. Consent for linkage to administrative health records was obtained in 2009. A likelihood score (Lscore) is computed, relating symptoms to asthma diagnosis. Self-reported asthma and the Lscore are examined against duration of pesticide exposures. Of the 10,767 farmers listed, 2426 were still living, had farmed grain and were interviewed; 1371 were re-contacted and matched to health records. After allowance for confounders, years of exposure to phenoxy compounds are related to self-reported asthma and Lscore. Compared to no exposure, the adjusted odds ratios (95% Confidence Intervals for self-reported asthma for short, medium and long exposure to phenoxy compounds are 1.29 (0.66–2.52), 2.52 (1.25–5.09), and 3.18 (1.54–6.58), and for Lscore are 1.19 (0.91–1.55), 1.50 (1.13–1.99), and 1.58 (1.18–2.12). We conclude that lifetime exposure to phenoxy herbicides is associated with an increased risk of asthma.

## 1. Introduction

Grain farmers in Alberta, as elsewhere in the world, make substantial use of pesticides including herbicides and seed treatments. In Alberta, in 2005, herbicides were applied to 6.4 million hectares of farmland, fungicides to 653,146 hectares and insecticides to 493,226 hectares [1]. Details of pesticide use in Alberta, and on the commodities to which these are applied are published annually [2]. The lungs of farmers using pesticides may be exposed through inhalation during mixing or spraying of the pesticide, or through pesticide-contaminated soil-dust. The present study was set up to investigate the health of farmers, who would have been exposed over many years, and where effects, if any, reflected cumulative damage rather than an acute manifestation of recent exposure. An initial interest of the group was in the effects of organophosphates and other pesticides on mental ill-health in these farmers [3]. This required detailed information on lifetime use of all major pesticides. Such a lifetime exposure assessment is challenging and few studies have attempted it [4,5]. Studies of pesticide exposure in several countries, covering a variety of climates, types of farming and pesticide composition suggest relationships between pesticide exposure and wheeze, asthma or other obstructive lung disease: these include Kenya [6], Australia [7], Costa Rica [8], The Netherlands [9], Iowa and North Carolina in the United States [10], and in Canada the Province of Saskatchewan [11]. An earlier study in Alberta of predominantly grain farmers found an increase in obstructive lung disease and poorer lung function with self-reports of dust exposure, but did not consider the role of pesticides [12]. A systematic review in 2014 concluded that, while there was sufficient evidence to suggest that exposure to pesticides may be associated with a greater prevalence of asthma and wheeze, the evidence was insufficient to identify specific causal pesticide groups [13]. As such, it seemed important to use the lifetime exposure assessment of major classes of pesticides carried out for the study of mental health [3] to characterise more fully any relation of pesticide exposure to asthma in the Alberta farming community. 

## 2. Methods and Materials

Two cohorts of farmers believed to have grown grain commercially were identified. Cohort A was of farmers (mainly but not necessarily grain farmers) who had been registered with a provincial farming organization in 1983. Those in Cohort B were grain farmers who, in 2002, were on a list of those who had signalled to the provincial department of agriculture that they might be approached for research studies. A letter was sent to the address of each farmer, as given in the 1983 or research panel lists, asking him (or her) to return a consent form if willing to take part in the study. One reminder was sent. For cohort A, the consent form included a question to confirm whether he or she had ever been a grain farmer. Farmers in both cohorts were asked to supply telephone numbers and to indicate the best time for a telephone interview. Where a farmer appeared in both cohorts, they were approached only once and included with cohort A for the statistical analysis.

### 2.1. Measurement of Health Outcome

#### 2.1.1. Self-Report

During the telephone interview, the farmer completed a checklist of 30 chronic conditions including tuberculosis, other lung disease and allergy, asking for each whether “a doctor had ever told you that you had any of the following medical problems”. If the subject said “yes” to any condition, they were asked the year in which they were first diagnosed with the condition. Any comments volunteered were noted (for example, the agent to which they thought they were allergic). The interviewer then asked questions selected from the American Thoracic Society (ATS) respiratory questionnaire [13] relating to wheezing or asthma. These were:

Does your chest ever sound wheezy or whistling

(a) when you have a cold
(b) occasionally apart from colds
(c) most days or nights?

If “yes” to any, for how many years this has been present?

Have you ever had an attack of wheezing that has made you feel short of breath?

Has a doctor ever told you that you have asthma? 

If “yes”, how old were you when this was first diagnosed?

These are D2–D4 in the study questionnaire attached as Supplementary Materials.

A positive answer to the question “has a doctor ever told you that you have asthma” is referred below as “self-reported asthma”.

#### 2.1.2. Medical Records

In 2009, the farmers taking part in the initial study were re-contacted to ask their consent for their questionnaire data to be linked to information extracted from the Alberta administrative health database. In Alberta, all physician consultations are paid through the provincial heath service, with the physician recording diagnoses when submitting billings to Albert Health. Diagnoses of asthma (ICD-9 code 493) were extracted to the date of questionnaire completion in March 2002 for all in-patient (from May 1994), out-patient (from September 1998) or physician billing (from May 1994).

### 2.2. Measurement of Confounders

The interviewer collected information on demographic factors including age and sex, total years in farming, current activity (hours working as a grain farmer) and exposure to pesticides in the previous month. Smoking history was collected by postal questionnaire in a second contact in May 2004. Where a response was received from relatives of a farmer who had died since the March 2002 questionnaire and the response included smoking information for the participant, this proxy information was used.

### 2.3. Assessment of Exposure

When the initial consent form was received in March 2002, a further letter was sent enclosing three lists giving the trade names of insecticides (26 names), herbicides (48 names), and seed treatments (25 names) as shown as questions C7, C14 and C21 in the questionnaire included as Supplementary Material. The farmer was asked to review these before the interview to identify products they had ever used and the years in which they had done so. Active ingredients for each named pesticide (either in the pre-circulated lists or volunteered at the interview) were identified from the crop protection guides published annually by Alberta Agriculture for the period 1981–2003 [14]. Pesticides used prior to this or not listed were classified in discussion with Alberta Agriculture. This information was collected in a database created for the project, which reflected changes in product formulation during the period studied as reflected in the annual crop protection guides. Seven classes of active ingredients in pesticides were identified for the present analysis: phenoxy acids, organochlorines, organophosphates, pyrethroids, carbamates, thiocarbamates and dithiocarbamates. The use of each class of pesticide was recorded for each year from the respondent’s first year in farming (since age 18 years) to the date of the interview in March 2002. Custom application by someone other than the subject was excluded. A conservative approach to estimating exposure was adopted. Years using a type of pesticide were included only where the active ingredients could be definitively assigned from the trade name and dates reported. Where it could not, the years using the pesticide were ignored and coded as “not used”. Where there was doubt about the length of use of a particular pesticide type, the minimum estimate was used. The total years in which there was any use of the pesticide type (“total years of use”) for each class of pesticide was divided into tertiles for those reporting any exposure and referred to descriptively as “no”, “short”, “medium” and “long” duration. 

### 2.4. Ethical Approval

The study was considered and approved by the Ethics Board of the University of Alberta (Pro00004746).

## 3. Statistical Methods

Self-reported physician-diagnosed asthma was examined against tertiles of exposure years within each chemical class of pesticide individually, computing univariate χ^2^ tests for trend. Diagnoses of asthma found in the Alberta Health administrative database for those contacted in 2009 was used to supplement self-reports of asthma. An outcome score was computed, labelled here as the Lscore, that reflected the likelihood of a recorded diagnosis of asthma. To compute the Lscore we used logistic regression in Stata with a diagnosis of asthma in the Alberta Health administrative database as the dependent variable. Robust standard errors were used to allow for violation of model assumptions in logistic regression. The model initially included all questions (given above) relating to wheeze and asthma but retained only those that, on a Wald test, improved the prediction. The possible influence of age, sex, smoking and cohort of origin on diagnostic reporting was examined by adding each in turn to the model. As many farmers who had completed the March 2002 questionnaire had died or moved away from the farm and could not be contacted in 2009 to give consent to data linkage, Lscores were assigned for these farmers also using the same predictive model. Likelihood was calculated using the coefficients from the logistic regression, weighted by the presence (1) or absence (0) of each symptom. The inverse logit of the prediction score gave the probability (Lscore). The Lscore was then used as the dependent variable in a series of multivariable analyses. As the Lscore was a fractional response (the probability of an asthma diagnosis in health records), GLM with a logit link and with robust standard errors was used. The same model was used for the binary outcome of self-reported asthma [15]. 

The analyses included exposure estimates for all seven exposure groups (as categorical scores based on tertiles of increasing duration of use) and, as potential confounders, age, sex and: (i) work status (not working as a farmer, working as a farmer but not with grain, grain farming <20 h/week, grain farming 20 h/week or more); (ii) exposure to pesticides in the last month; (iii) smoking (current, ex-smoker, never smoker, unknown); and (iv) a factor reflecting whether they had been matched to Alberta health records. Further analyses considered the effect of adding to the models total years farming (since grain dust itself may be related to asthma), self-reported allergy, a self-reported history of “pesticide poisoning” and the cohort of origin. The final model including all exposures, confounders and these additional factors, was re-run excluding those with asthma diagnosed at less than 18 years of age. Stratification was used to further explore the role of allergy and the effect of recruitment through two cohorts with different characteristics.

## 4. Results

In Cohort A, 5986 names were listed as members of the farm association in 1983. In March 2002, contact was made with 2493 of these farmers (41.7%), 1348 of whom (54.1%) confirmed that they had farmed grain commercially and agreed to answer the questionnaire. In Cohort B, there were 4571 names provided; of these, 2503 responded to the contact (54.8%) and 1078 (43.1%) had farmed grain and were willing to be interviewed. 

Use of pesticides (herbicides, insecticides, or seed treatments) was almost universal in both cohorts: all but nine in cohort A (<1.0%) and 61 in cohort B (6%) reported that they had used pesticides at some point. Only three of those who used pesticides failed to identify any of them.

Smoking status was obtained for 2241 (92.4%), including 70 proxy responses, in May 2004. In 2009, 1371 farmers (732 from cohort A and 639 from cohort B) were contacted, gave consent to data linkage and were successfully matched to the Alberta administrative health record. Demographic, self-reported asthma and related data for the two cohorts and those matched or not to the health record are shown in Table 1. The two cohorts differed on every factor considered other than reports of pesticide poisoning and asthma. Those in Cohort B were younger, included more women and were more likely to be working and to report allergies. Those in Cohort B were more likely to report recent exposures. There were also important differences between those matched or not to their health record. Those not matched were older, less likely to be working and less likely to have been exposed to pesticides in the month before the questionnaire. A greater proportion had no smoking data (as they had died or been lost to trace before this was collected) and more reported that they had asthma.

The estimated duration of use of pesticides in the seven chemical groups is shown by self-reported asthma in Table 2. Phenoxy compounds were the only chemical group related with a linear trend to self-reported asthma in this univariate analysis. No difference was found in the duration of use of phenoxy compounds in those matched to health records (mean 25.9 years SD 14.7) and those unmatched (mean 25.3 years SD 16.1).

When the questions relating to asthma were used to predict a diagnosis of asthma in the physician billing administrative records (health records) for the 1371 successfully matched, only four of the questions (wheezing with cold, wheezing without cold, made short of breath by wheezing and “a doctor had said asthma”) contributed to the model (Table 3). Age, sex, smoking, cohort and self-reported allergy were added sequentially to this model and none added significantly (data not shown). The Lscores for all subjects, regardless of whether they had been included in the analysis in Table 3, are shown, by self-report combinations, in Table 4. Those not matched had a higher mean Lscore (0.10, SD = 0.14) than those matched (0.08, SD = 0.13) *p* < 0.001, reflecting the higher proportion with self-reported asthma.

A multivariable model allowing for potential confounders and all pesticide groups is given in Table 5 for the two outcomes, self-reported asthma and Lscore. The increase in odds ratio (OR) with higher tertile category for phenoxy herbicides is seen for both outcomes, although with smaller estimates and narrower confidence intervals with the Lscore. Smoking and currently working in farming other than grain farming was associated with a higher Lscore, but no other pesticide group showed increased risk. Those linked to the health records were, as expected from the univariate analyses, less likely to report asthma and had a lower Lscore.

Total years farming, concurrent reports of allergy and self-reports of pesticide poisoning were entered sequentially into the model. The addition of total years of farming did little to change the estimated ORs for phenoxy compounds (Table 6). Similarly, although self-reported allergy was strongly related to both self-reported asthma and the Lscore, its inclusion had little effect on the phenoxy estimates. The inclusion of the cohort of origin as a factor was not significant overall for either outcome measure (OR = 0.85, 95% Confidence Interval (CI) 0.60–1.20 for asthma; OR = 0.91, 95% CI = 0.78–1.06 for Lscore). A self-report of pesticide poisoning earlier in life was unrelated to self-reported asthma (OR = 1.26, 95% CI 0.92–1.73) after adjustment for other factors but was associated with higher risk using the Lscore (OR = 1.34, 95% CI 1.17–1.53). 

Overall, 24% of the farmers reported that a doctor had told them that they had allergies. When the model was run separately for those with and without allergy, the ORs for phenoxy compounds with self-reported asthma were higher in those not reporting allergy. With the Lscore the ORs were somewhat higher in those reporting an allergy, although with the reduced sample size the ORs in this group did not exclude 1.0. Stratification by cohort suggested stronger effects in the older cohort.

Of the 202 farmers self-reporting asthma, 46 said this was first diagnosed under the age of 18 years. Table 6 shows the ORs for phenoxy compounds for the full sample, adjusting for all other factors and then for the same model with those farmers with early diagnosed asthmas excluded. The ORs for phenoxy compounds were reduced but a clear increase in risk of both outcomes was still apparent.

## 5. Discussion

This study examined the effects of long term exposure to pesticides used in grain farming on the respiratory health of farmers in Alberta, a province with a climate that ensures that insecticides (such as organophosphates) are not routinely in high use but with a sustained use of herbicides We aimed to characterize the types of pesticides, if any, related to asthma in our cohorts exposed over many years and to test an observation from the neighbouring grain producing province of Saskatchewan that self-reported asthma was related to carbamate insecticide exposure [11]. We did not find evidence of that in this study, but we did find effects on asthma reporting of exposure to phenoxy compounds, a herbicide active ingredient widely used for many years but studied mainly for carcinogenic effects. Review of the literature on non-malignant morbidity from long term exposures to specific chemical groups of pesticides found few studies of any size other than the US Agricultural Health Study (AHS), which has published results largely by trade name. In the recent publication [10] on wheeze, results were presented by chemical group allowing more direct comparison with the results of the present study. The AHS reported an increase in wheeze [10] and self-reported asthma [16] with higher exposure to specific herbicides and insecticides, including 2,4-D, a common phenoxy herbicide widely used by Alberta grain farmers. In the AHS study, wheeze in the last year with 2,4-D appeared to be limited to those who told the study team they had a history of physician diagnosed hay fever [10], while the relation between life time exposure to 2,4-D and self-reported asthma was only found for those reporting physician diagnosed hay fever or eczema [16]. In the study reported here, stratifying by report of physician diagnosed allergy did not suggest that the increased risk of asthma with phenoxy compounds was limited to those with a history of allergy, and so did not fully replicate the AHS finding. However, given the likelihood of an irritant mechanism [17], in addition to any allergic (IgE mediated) mechanism as proposed by the AHS group, it is perhaps sufficient to conclude, considering results both from the AHS and the present study, that phenoxy compounds are capable of producing wheeze and, with prolonged use, asthma.

This study’s strength is that it provides information on the effects of long term exposure to different classes of pesticide in cohorts of farmers exposed over many years. Recall (or recognition) of pesticide trade names was good (in that most farmers could name the pesticides used) and enabled identification of the active ingredient in the substantial majority of reported exposures. Allocation of reported pesticides to exposure groups was carried out by computer linkage to a pesticide database devised for this project. It was wholly independent of the health status of the participant. We were conservative in only allocating to exposure groups years in which we were confident that a product was used, which implies that misclassification of exposure would largely be underestimation. Use of duration alone was inevitably correlated with age and number of years as a grain farmer. However, total years in farming did not change the relation of asthma to phenoxy herbicides, making confounding by exposure to general farm dust [12] or grain handling [18] less likely as an explanation for the results from this study. Only 18 of the farmers reporting allergy volunteered grain dust as a cause: four of these also reported asthma, three of whom mentioned other allergens (grass, pollen, mold, dust, animal dander) as well as grain dust. It remains possible that duration of phenoxy compound use is in itself confounded by some unknown and unmeasured exposure or is acting as a proxy for some other pesticide not estimated here or estimated with much greater error.

The study also has weaknesses. The tracing and response rate was low: many had died or moved, and for cohort A the ethics board put restrictions on active tracing. The cohort (B) of “active” grain farmers comprised those who had indicated, when applying for tax credits, that they were willing to be approached for investigations: they may have been quite unrepresentative of all grain farmers. Of necessity, the study is confined to survivors.

An exposure metric reflecting only years with use is clearly not optimal. Ideally, we would have information on practices likely to result in high intensity of exposure and on the use of protective equipment as well as the total hours of use in each year. However, because of the many years of exposure and the age of the respondents it would have been unrealistic to attempt to get from them details for each year on intensity related practices and personal protection as used in the original AHS exposure estimates, or to collect meaningful biological samples to validate this [19,20]. We were concerned that any misclassification resulting from poor recall or incomplete information should be non-differential. In particular we wanted to avoid the possibility that those who believed that their respiratory ill-health was caused by exposure to pesticides would over-report. Simply classifying by any use is likely to have minimized such an effect, and the specificity of the phenoxy compounds, rather than any other (or all) pesticide types, suggests that there was no such general over-reporting in those who were unwell.

In carrying out the analysis reported here, we were particularly concerned about misclassification in the outcome variable, the diagnosis of asthma. Since the study is set in Alberta, with access to administrative health records, we hoped to go beyond the practice in other large community-based studies of farmers of relying on self-report: few studies have been able to access lung function testing and these have not generally been used to assess the effect of specific pesticides. Because of the relatively incomplete (61% matched) and unrepresentative matching to health records (with those matched being younger, less likely to smoke or to self-report asthma) use of administrative heath records alone proved not useful and we elected instead to use both self-report of asthma and the likelihood score derived from the wider set of health questions and the health records of asthma. Such health records, while independent of any biased response tendency in the study participant, are a very imperfect reflection of asthma as rigorously diagnosed. Asthma is recognised to be complex to diagnose in the elderly, and as a result underdiagnosed and treated [21]. Moreover, diagnoses reported for billing purposes may be made more hastily than for treatment or research, and represent only an afterthought to the main reason for the consultation. While use of the physician diagnosis of asthma in the health records allowed us to produce a score that compliments self-report, use of the resulting scores will tend to bias estimates of effect towards the null, with overly narrow confidence intervals [22,23]. However, use of the two approaches to asthma assessment gives some confidence that over-reporting of asthma or exposures by those who believe the two to be linked, may not in itself fully account for the relationship to phenoxy compounds reported here.

This study underlines the need to follow a cohort over many years to assess the chronic effects of lifetime exposure; the farmers in these analyses had been working for, on average, close to 40 years and many for considerably longer. The study may be unique in considering respiratory ill-health in farmers so late in life.

## 6. Conclusions

This study has considered the effect of major classes of pesticides, encountered over many years, on the respiratory health of grain farmers working in a northerly province of Canada and concluded that there is evidence for an increased risk of asthma with repeated exposure to phenoxy compounds. For some other classes of pesticide, such as pyrethroids, use was too infrequent for any strong conclusion to be reached, and it may be that in other types of farming, or in other climates, the pattern of risks would differ. For the protection of farmers using phenoxy compounds, it is important to understand the mechanism of any increased risk of asthma. Lung irritation is recognized to be an acute effect of inhalation of this class of herbicides, but it is uncertain whether the effects seen here after many years of use can best be considered a form of reactive airways dysfunction syndrome (RADS) or whether, as previously suggested [10,16], there is an allergic component. Either way, control of exposure is paramount. The findings of the study strongly support user-education programs to improve safe-handling of these widely used compounds.

## Figures and Tables

**Table 1:** Demographic, work and self-reported exposure, allergy and asthma by cohort and record linkage.

Questionnaire Response	Cohort					Linked to Provincial Health Record?					Overall	
	1983 List of Farming Association		2002 List of Grain Farmers		Difference	Yes		No		Difference		
	*N*	%	*N*	%	*p*	*N*	%	*N*	%	*p*	*N*	%
Sex												
Male	1333	98.9	946	87.8	≤0.001	1288	93.9	991	93.9	0.99	2279	93.9
Age (years)												
20–50	111	8.2	368	34.2	≤0.001	285	20.8	194	18.4	≤0.001	479	19.8
51–60	273	20.3	297	27.6		378	27.6	192	18.2		570	23.5
61–70	416	30.9	265	24.6		411	30.0	270	25.6		681	28.1
71–98	548	40.7	146	13.6		297	21.7	397	37.7		694	28.6
Smoking												
never	669	49.6	551	51.1	≤0.001	761	55.5	459	43.5	≤0.001	1220	50.3
current	70	5.2	103	9.6		88	6.4	85	8.1		173	7.1
ex-smoker	483	35.8	365	33.9		515	37.6	333	31.6		848	35.0
unknown	126	9.3	59	5.5		7	0.5	178	16.9		185	7.6
Workstate now												
grain farmer					≤0.001					≤0.001		
≥20 h/week	486	36.1	594	55.1		684	49.9	396	37.5		1080	44.5
<20 h/week	122	9.1	159	14.7		170	12.4	111	10.5		281	11.6
farmer, not grain	138	10.2	201	18.6		193	14.1	146	13.8		339	14.0
not working as farmer	602	44.7	124	11.5		324	23.6	402	38.1		726	29.9
Exposure to pesticides in last month?												
Yes	317	23.5	89	8.3	≤0.001	267	19.5	139	13.2	≤0.001	406	16.7
Ever had symptoms of pesticide poising?												
Yes	439	32.6	337	31.3	0.486	447	32.6	329	31.2	0.467	776	32.0
Doctor ever said you have allergies?												
Yes	283	21.0	300	27.8	≤0.001	339	24.7	244	23.1	0.361	583	24.0
Doctor ever said you have asthma? *												
Yes	109	8.1	93	8.7	0.621	92	6.7	110	10.4	0.001	202	8.3
Overall	1348	100	1078	100	-	1371	100	1055	100	-	2426	100

* Four people did not respond to the asthma question and have been excluded from subsequent analyses.

**Table 2:** Estimated duration of exposure by chemical group and self-reported asthma.

Chemical Group	Years	Distribution of Exposure		Self-Reported Asthma		χ^2^ for Trend
	Exposed	*N*	%	*N*	%	*p*
Phenoxy compounds	0	249	10.3	17	6.8	0.012
	1–22	732	30.2	48	6.6	
	23–34	700	28.9	62	8.9	
	≥35	741	30.6	75	10.1	
Organochlorines	0	931	38.4	81	8.7	0.810
	1–13	500	20.6	35	7.0	
	14–27	509	21.0	42	8.3	
	≥28	482	19.9	44	9.1	
Organophosphates	0	1347	55.6	109	8.1	0.337
	1–5	352	14.5	27	7.7	
	6–19	339	14.0	28	8.3	
	≥20	384	15.9	38	9.9	
Pyrethroids	0	2038	84.1	168	8.2	0.971
	1	134	5.5	14	10.4	
	2–4	136	5.6	12	8.8	
	≥5	114	4.7	8	7.0	
Carbamates	0	1830	75.6	158	8.6	0.639
	1–3	216	8.9	15	6.9	
	4–9	181	7.5	11	6.1	
	≥10	195	8.1	18	9.2	
Thiocarbamates	0	875	36.1	81	9.3	0.550
	1–6	485	20.0	33	6.8	
	7–19	498	20.6	42	8.4	
	≥20	564	23.3	46	8.2	
Dithiocarbamates	0	909	37.5	75	8.3	0.388
	1–6	521	21.5	37	7.1	
	7–19	495	20.4	43	8.7	
	≥20	497	20.5	47	9.5	
Overall		2422	100	202	8.3	-

**Table 3:** Prediction of asthma diagnosis in physician administrative data from self-reported ill-health in the linked sample (*N* = 1367).

Health Response	Reporting “Yes”		Odds Ratio	95% CI	Regression Coefficient
	*N*	%			
Does your chest ever sound wheezy or whistling?					
When you have a cold?	394	28.8	2.14	1.29–3.56	0.761
Occasionally apart from cold?	201	14.7	2.35	1.36–4.05	0.854
Have you had an attack of wheezing that has made you feel short of breath?	178	13.0	2.63	1.56–4.42	0.967
Has a doctor ever told you that you have asthma?	92	6.7	5.11	2.89–9.04	1.631
Regression constant					−3.443

**Table 4:** Predicted likelihood of asthma record in administrative health records calculated for each combination of symptoms from the self-report questionnaire (*N* = 2422).

Wheeze with Cold	Occasional Wheeze without a Cold	Attack of Wheeze Causing Shortness of Breath	Told Have Asthma by MD	Predicted Likelihood of Asthma Record	Number of Observations in Whole Sample	% in Whole Sample
0	0	0	0	0.031	1421	58.7
0	0	0	1	0.140	26	1.1
0	0	1	0	0.078	71	2.9
0	1	0	0	0.070	76	3.1
1	0	0	0	0.064	376	15.5
1	0	0	1	0.259	23	1.0
1	0	1	0	0.152	64	2.6
1	1	0	0	0.138	117	4.8
0	0	1	1	0.300	16	0.7
0	1	0	1	0.277	8	0.3
0	1	1	0	0.165	28	1.2
0	1	1	1	0.502	15	0.6
1	0	1	1	0.479	18	0.7
1	1	0	1	0.451	26	1.1
1	1	1	0	0.297	67	2.8
1	1	1	1	0.683	70	2.9
Overall					2422	100.0

**Table 5:** Multivariable regression of exposures and potential confounders on self-reported asthma and Lscore (likelihood of asthma recorded in health records) (*N* = 2420 *).

Pesticide Durations and Potential Confounders	Asthma		Lscore	
	OR	95% CI	OR	95% CI
Age	1	0.98–1.01	1	0.99–1.00
Sex				
Male	0.66	0.33–1.30	0.79	0.58–1.06
Workstate				
Full time grain	1	-	1	-
Part time grain	1.31	0.82–2.10	1.16	0.93–1.44
Other farmer	1.30	0.83–2.04	1.32	1.08–1.62
Not working as farmer	0.89	0.59–1.34	0.93	0.78–1.11
Exposure to pesticides last month	1.07	0.71–1.61	1.02	0.84–1.23
Smoking				
Never	1	-	1	-
Current	1.45	0.83–2.51	1.42	1.12–1.79
Ex-smoker	1.23	0.89–1.69	1.21	1.05–1.40
Unknown	1.36	0.79–2.35	1.34	1.03–1.75
Linked to health records	0.63	0.46–0.85	0.82	0.72–0.94
Years using				
Phenoxy				
None	1	-	1	-
1–22	1.47	0.75–2.88	1.24	0.94–1.62
23–34	2.22	1.11–4.46	1.45	1.09–1.94
≥35	2.48	1.23–4.99	1.48	1.09–1.99
Organochlorines				
None	1	-	1	-
1–13	0.78	0.46–1.33	1.05	0.83–1.34
14–27	0.77	0.42–1.40	0.96	0.74–1.25
≥28	0.82	0.47–1.43	1.06	0.82–1.36
Organophosphates				
None	1	-	1	-
1–5	1.05	0.65–1.70	0.93	0.75–1.14
6–19	1.13	0.72–1.76	1.08	0.89–1.32
≥20	1.21	0.79–1.85	1.02	0.84–1.24
Pyrethroids				
None	1	-	1	-
1	1.47	0.78–2.78	1.22	0.89–1.68
2–4	1.25	0.64–2.44	1.05	0.77–1.42
≥5	0.79	0.36–1.74	0.89	0.65–1.21
Carbamates				
None	1	-	1	-
1–3	0.72	0.39–1.31	1.03	0.82–1.31
4–9	0.61	0.31–1.22	0.85	0.66–1.08
≥10	0.99	0.55–1.77	1.09	0.83–1.45
Thiocarbamates				
None	1	-	1	-
1–6	0.72	0.46–1.12	0.84	0.70–1.02
7–19	0.83	0.54–1.28	0.90	0.74–1.09
≥20	0.70	0.46–1.06	0.91	0.75–1.10
Dithiocarbamates				
None	1	-	1	-
1–6	1.03	0.61–1.76	1.08	0.85–1.37
7–19	1.35	0.72–2.52	1.11	0.84–1.47
≥20	1.29	0.74–2.26	1.04	0.81–1.34

* Two participants with missing age were omitted. Adjusted for all other factors shown.

**Table 6:** Effect on risk estimates * for phenoxy compounds with self-reported asthma and Lscore of adding further factors and of stratification (*N* = 2420).

Further Factor Added	Years Using Phenoxy Compounds								Total Years		Allergy	
	None		1–22		23–34		≥35		In Farming			
	OR	95% CI	OR	95% CI	OR	95% CI	OR	95% CI	OR	95% CI	OR	95% CI
Total years in farming												
Asthma	1	-	1.42	0.73–2.77	2.35	1.17–4.71	2.83	1.38–5.81	0.98	0.96–1.00	-	-
L Score	1	-	1.23	0.94–1.62	1.47	1.10–1.96	1.52	1.12–2.06	0.99	0.98–1.00	-	-
Allergy self-report												
Asthma	1	-	1.35	0.69–2.64	2.38	1.18–4.79	2.76	1.36–5.61	-	-	4.82	3.55–6.55
L Score	1	-	1.19	0.91–1.55	1.48	1.12–1.96	1.53	1.14–2.05	-	-	2.33	2.03–2.67
Stratified by allergy												
No report (*N* = 1838)												
Asthma	1	-	1.59	0.47–5.32	3.05	0.92–10.13	3.68	1.10–12.28	-	-	-	-
L Score	1	-	1.15	0.84–1.57	1.40	1.01–1.94	1.51	1.07–2.12	-	-	-	-
Some report (*N* = 582)												
Asthma	1	-	1.19	0.50–2.81	2.21	0.86–5.67	2.38	0.89–6.35	-	-	-	-
L Score	1	-	1.23	0.76–1.98	1.68	0.99–2.85	1.63	0.93–2.86	-	-	-	-
Stratified by cohort												
Early cohort (*N* = 1347)												
Asthma	1	-	2.31	0.62–8.53	3.34	0.90–12.34	4.15	1.12–15.36	-	-	-	-
L Score	1	-	1.61	1.04–2.47	1.89	1.24–2.89	1.90	1.24–2.91	-	-	-	-
Later cohort (*N* = 1073)												
Asthma	1	-	1.01	0.43–2.36	1.73	0.71–4.25	1.49	0.57–3.88	-	-	-	-
L Score	1	-	1.07	0.76–1.51	1.28	0.87–1.88	1.27	0.82–1.95	-	-	-	-
All factors **												
Asthma	1	-	1.29	0.66–2.52	2.52	1.25–5.09	3.18	1.54–6.58	0.97	0.95–1.00	4.82	3.51–6.62
L Score	1	-	1.19	0.91–1.55	1.50	1.13–1.99	1.58	1.18–2.12	0.99	0.98–1.00	2.26	1.97–2.61
All factors excluding those diagnosed with asthma <18 years old (*N* = 2373)												
Asthma	1	-	1.28	0.61–2.66	2.16	0.99–4.71	2.63	1.17–5.90	0.98	0.96–1.00	3.87	2.72–5.52
L Score	1	-	1.18	0.90–1.53	1.38	1.04–1.83	1.43	1.07–1.92	1.00	0.99–1.01	2.01	1.74–2.32

* Adjusted for all factors in Table 5. ** All factors in Table 5 plus total years farming, report of allergy, cohort and report of pesticide poisoning.

## Supplementary Materials

The following are available online at http://www.mdpi.com/1660-4601/15/3/526/s1, Pesticides and asthma full questionnaire
