# Supplementary material for: Pesticide Use and Asthma in Alberta Grain Farmers
Source: Int J Environ Res Public Health. 2018 Mar 15;15(3):526. doi: 10.3390/ijerph15030526 (PMC5877071; doi:10.3390/ijerph15030526)
Supplement: Supplementary file 1 [file ijerph-15-00526-s001.pdf]

ID No.: ________________________

**Pesticides and Asthma Full Questionnaire**

Name of Farmer: ______________________________________________

Address: ______________________________________________________

______________________________________________________

Telephone Number: (_______) _________________________________

*********************************************************************************************

Name of Interviewer: ______________________________________

| Date of Contact | Time of Contact | Outcome of Contact |
|---|---|---|
| | | |
| | | |
| | | |
| | | |
| | | |
| | | |
| | | |
| | | |
| | | |

DATE OF INTERVIEW: ______________________________________

TIME INTERVIEW STARTED: __________________________________

TIME INTERVIEW ENDED: ____________________________________

## Interviewer Comment Sheet

**INTRODUCTION**
Hello Mr/Mrs____________. My name is ____________ and I am calling from the University of Alberta. You may remember, we mailed you a letter and information sheet about our study on Pesticide Use and the Health of Farmers a few weeks ago. You said you would be willing to take part and we would like to carry out the telephone interview with you now. This will take about 20 minutes. Is this a good time for you? Have you got the lists of pesticides previously sent with you now? I would just like to clarify that throughout the interview, when I say pesticides I am referring to insecticides, herbicides, and fungicides/seed treatments.

**Section A – Establishment of current status**

A1. Are you **now** working as a farmer?............................................... ☐ Yes
☐ No

A1a. Have you ever worked **as** a farmer? ........................................... ☐ Yes
☐ No

A1b. **If A1 or A1a is no**, *apologize and end the interview*

A2. If ever worked as a farmer – have you ever worked as a farmer who grows grain and sells it commercially? .................................................................. ☐ Yes
☐ No

A2a. **If no**, what sort of farming have you mostly done? (*Record the answer below then apologize and end the interview*)

______________________________________________________________

A3. Are you working now as a farmer who grows grain and sells it commercially?.................................................................... ☐ Yes
☐ No

A3a. **If no**, when did you last grow grain and sell it commercially? _______________

______________________________________________________________

****If A3 is no,** *go to question B1.*******

A4. If working as a grain farmer now, how many hours a week do you spend actively involved in growing and selling grain?

______________________________________________________________

A4a. **If less than 20 hours per week**, in what year did you last work for as much as 20 hours a week in growing and selling grain?

______________________________________________________________

**Section B**

**Interviewer:** Please document gender: Female ☐ Male ☐

**Questions B1-B6 – Ask all**

B1. How old were you on your last birthday? ______________________________

B2. What grade did you complete in school? ______________________________

______________________________________________________

B3. Have you ever attended lectures or taken courses of instruction dealing with the safe and proper use of pesticides?.................................................... ☐ Yes
☐ No

B3a. **If yes**, in what year was the instruction taken? ______________________

B4. Have you attended university or taken university degree credit courses?.. ☐ Yes
☐ No

B4a. **If yes**, how many years or courses have you completed? __________ years,

______________________________________________________

B5. How many years have you been working in agriculture since the age of 18 (do not include years of education as experience)?

______________________________________________________

B6. How many years have you been a farmer or farm operator since the age of 18 (with actual decision making responsibilities)?

______________________________________________________

**Questions B7-B10 – ask active farmers only**
**In this section - treat those currently working for 20 hours a week or less as not active**

****(If inactive farmer (if A3 is no or if A4a is less than 20 hours a week ) say: The next few questions are about the use of pesticides. Once again, I would like to remind you that when I mention pesticides, I am referring to insecticides, herbicides, and fungicides/seed treatments. – then go to question B11****

B7. What is the total area of all land you currently operate (include all land owned, leased, or rented)?

______________________________________________________

______________________________________________________

B8. Approximately how many acres of the land that you currently farm was treated with herbicide in 2002?

________________________________________

**** The next few questions are about the use of pesticides. Once again, I would like to remind you that when I mention pesticides, I am referring to insecticides, herbicides, and fungicides/seed treatments.****

B9. In 2002, what percentage of your pesticide application was done by a custom applicator (include the work done by a neighbor or friend on an exchange bases)?

________________________________________

B10. During 2002 spraying season, did you experience what you believe to be symptoms of pesticide poisoning?............................................................ ☐ Yes
☐ No

B10a. **If yes**, what symptoms did you experience?

________________________________________

________________________________________

________________________________________

B10b. How long were these symptoms experienced?

________________________________________

B10c. What pesticide or pesticides do you believe produced these symptoms?

________________________________________

________________________________________

**Questions B11-B15 – Ask all**

B11. In previous years, have you experienced what you believed to be symptoms of pesticide poisoning?....................................................................... ☐ Yes
☐ No
☐ Not Sure

B11a. On how many separate occasions did you experience these symptoms?

________________________________________

B11b. What pesticide or pesticides do you believe caused these symptoms?

____________________________________________________________

____________________________________________________________

B12. Have you ever sought medical attention as the result of exposure to pesticides? .................................................................... ☐ Yes
☐ No

B12a. **If yes**, when you sought medical attention, did you receive what you consider to be adequate aid? ............................................................ ☐ Yes
☐ No
☐ Not Sure

B13. Was there any particular reason for **NOT** seeking medical attention?

____________________________________________________________

****If medical attention NOT sought because of never having symptoms of pesticide poisoning (B11) or from exposure to pesticides (B12), tick box** .................................................................... ☐

B14. Do you believe that you have ever experienced long-term, lingering, or chronic health problems due to pesticide exposure over the years? ......................... ☐ Yes
☐ No
☐ Not Sure

B14a. **If yes**, what health problems have you experienced?

____________________________________________________________

____________________________________________________________

____________________________________________________________

B14b. Are you currently experiencing them? ................................ ☐ Yes
☐ No
☐ Occasionally

B15. Have you ever experienced what you believe to be allergic reactions to pesticides? .................................................................... ☐ Yes
☐ No
☐ Not Sure

B15a. **If yes**, what pesticide or pesticides do you believe produced these allergic reactions?

____________________________________________________________

____________________________________________________________

**Questions B16-20 – ask active farmers only (*If inactive farmers* (if A3 is no or if A4a is less than 20 hours a week) – *go to question B21)***
**In this section - treat those currently working for 20 hours a week or less as not active**

B16. When you work with pesticides, do you wear any special clothing or pesticide safety equipment? ........................................................................ ☐ Yes
☐ No

B17. What do you usually wear when opening containers or mixing pesticides?

______________________________________________________________________

______________________________________________________________________

B18. What do you usually wear on the tractor when applying pesticides in the field?

______________________________________________________________________

______________________________________________________________________

B19. What do you usually wear when mixing a seed treatment with your seed?

______________________________________________________________________

______________________________________________________________________

B20. Do you feel that pesticide safety equipment is too uncomfortable?....... ☐ Yes
☐ No

**Question B 21 – Ask all**

B21. Do you feel that pesticides are particularly harmful to your health? ........................................................................... ☐ Yes
☐ No
☐ Not Sure

**Section C – Lifetime history of pesticide use** – **Ask all**

**In this section, I am going to ask you about insecticides, then about herbicides, and finally about seed treatments. Thinking first about insecticides;**

C1. Looking back over your years in farming, what was the first year you were involved in the application of **insecticides** to crops or stock?

________________________________________________________________

C2. What was the last year you were involved in the application of **insecticides** to crops or stock?

________________________________________________________________

C3. Do you remember any years in which you did **not** use these **insecticides**?

________________________________________________________________

C4. In the course of a typical year, how many days did you use these **insecticides**?

______________________days

C4a. For how many hours each day? __________________________ hours

C5. When using these **insecticides**, did you use any equipment or clothing to stop you from breathing them in or to stop them from getting on your skin?............. ☐ Yes
☐ No

C5a. **If yes**, what did you use (*be specific – eg latex/cloth/rubber gloves*)?

________________________________________________________________

________________________________________________________________

C6. Did you use **insecticides** in any other way around your house, yard (eg lawn), or garden? .................................................................... ☐ Yes
☐ No

C6a. What did you use? __________________________________________

C6b. For how many days per year? ____________________________________

C7. I will now go over the insecticide list previously sent to you. Please listen to this list of **insecticides** used in Alberta and identify what you use/used and when you used them. **Indicate first year used, last year used, and the number of years used in total.**

| Insecticide | Calendar Year(s) | Insecticide | Calendar Year(s) | Insecticide | Calendar Year(s) |
|---|---|---|---|---|---|
| Counter | F:<br>L:<br>T: | Dylox | F:<br>L:<br>T: | Premiere Plus Flowable | F:<br>L:<br>T: |
| Cygon 4E | F:<br>L:<br>T: | Foundation Seed Treatment | F:<br>L:<br>T: | Pyramin FL | F:<br>L:<br>T: |
| Cythion | F:<br>L:<br>T: | Furadan | F:<br>L:<br>T: | Pyrinex 480EC | F:<br>L:<br>T: |
| DB Green | F:<br>L:<br>T: | Hopper Stopper | F:<br>L:<br>T: | Sevin | F:<br>L:<br>T: |
| Decis 5EC | F:<br>L:<br>T: | Lagon 480 | F:<br>L:<br>T: | Thimet | F:<br>L:<br>T: |
| Decis Flowable | F:<br>L:<br>T: | Lindane | F:<br>L:<br>T: | Vitavax Dual Solution | F:<br>L:<br>T: |
| Diazinon | F:<br>L:<br>T: | Lorsban 4E | F:<br>L:<br>T: | Vitavax RS Dynaseal | F:<br>L:<br>T: |
| Dibrom | F:<br>L:<br>T: | Malathion 500 | F:<br>L:<br>T: | Vitavax RS Flowable | F:<br>L:<br>T: |
| Dimethoate 480 Systemic | F:<br>L:<br>T: | Matador 120EC | F:<br>L:<br>T: | | |

Others (please list any insecticides used not listed above)

| Insecticide | Calendar Year(s) | Insecticide | Calendar Year(s) | Insecticide | Calendar Year(s) |
|---|---|---|---|---|---|
| | | | | | |
| | | | | | |
| | | | | | |

C8. What was the first year you were involved in the treating the land with **herbicides**?

____________________________________________________________

C9. What was the last year you were involved in the treating the land with **herbicides**?

____________________________________________________________

C10. Do you remember any years in which you did **<u>not</u>** use these **herbicides**?

__________________________________________________________

C11. In the course of a typical year, how many days did you use these **herbicides**?

________________________days

C11a. For how many hours each day? ____________________________ hours

C12. When using these **herbicides**, did you use any equipment or clothing to stop you from breathing them in or to stop them from getting on your skin?.............. ☐ Yes
☐ No

C12a. **If yes**, what did you use (*be specific – eg latex/cloth/rubber gloves)*?

________________________________________________________

________________________________________________________

C13. Did you use **herbicides** in any other way around your house, yard (eg lawn), or garden? .......................................................................... ☐ Yes
☐ No

C13a. What did you use? ______________________________________________

C13b. For how many days per year? _______________________________________

C14. I will now go over the herbicide list previously sent to you. Please listen to this list of **herbicides** used in Alberta and identify what you use/used and when you used them. **Indicate first year used, last year used, and the number of years used in total.**

| Herbicide | Calendar Year(s) | Herbicide | Calendar Year(s) | Herbicide | Calendar Year(s) |
|---|---|---|---|---|---|
| 2, 4-D Amine | F:<br>L:<br>T: | Eptam 8-E | F:<br>L:<br>T: | Prestige | F:<br>L:<br>T: |
| 2, 4-D LV Ester | F:<br>L:<br>T: | Eradicane 8-E | F:<br>L:<br>T: | Prevail | F:<br>L:<br>T: |
| Achieve | F:<br>L:<br>T: | Estaprop | F:<br>L:<br>T: | Puma One Pass | F:<br>L:<br>T: |
| Advance | F:<br>L:<br>T: | Estasol | F:<br>L:<br>T: | Puma Super | F:<br>L:<br>T: |
| Assert | F:<br>L:<br>T: | Excel | F:<br>L:<br>T: | Renegade | F:<br>L:<br>T: |
| Attain | F:<br>L:<br>T: | Fortress | F:<br>L:<br>T: | Rival | F:<br>L:<br>T: |
| Avadex BW | F:<br>L:<br>T: | Glyfos | F:<br>L:<br>T: | Roundup Fast Forward | F:<br>L:<br>T: |
| Banvel | F:<br>L:<br>T: | Interprop | F:<br>L:<br>T: | Roundup Transorb | F:<br>L:<br>T: |
| Bonanza | F:<br>L:<br>T: | Laredo | F:<br>L:<br>T: | Rustler | F:<br>L:<br>T: |
| Buctril M | F:<br>L:<br>T: | Liberty | F:<br>L:<br>T: | Target | F:<br>L:<br>T: |
| Champion FM | F:<br>L:<br>T: | Lontrel 360 | F:<br>L:<br>T: | Thumper | F:<br>L:<br>T: |
| Curtail M | F:<br>L:<br>T: | MCPA-Amine | F:<br>L:<br>T: | Touchdown | F:<br>L:<br>T: |
| Dichlorprop D | F:<br>L:<br>T: | MCPA-Ester | F:<br>L:<br>T: | Treflan | F:<br>L:<br>T: |
| Diphenoprop | F:<br>L:<br>T: | MCPA-K | F:<br>L:<br>T: | Triumph FM | F:<br>L:<br>T: |
| Dyvel | F:<br>L:<br>T: | Pardner | F:<br>L:<br>T: | Turboprop 600 | F:<br>L:<br>T: |
| Edge | F:<br>L:<br>T: | Poast Ultra | F:<br>L:<br>T: | Victor | F:<br>L:<br>T: |

| Others (please list any herbicides used not listed above) | | | | | |
|---|---|---|---|---|---|
| **Herbicide** | **Calendar Year(s)** | **Herbicide** | **Calendar Year(s)** | **Herbicide** | **Calendar Year(s)** |
| | | | | | |
| | | | | | |
| | | | | | |

C15. What was the first year you were involved in **treating seed** (or handling seed that had already been treated)?

________________________________________________________

C16. What was the last year you were involved in **treating seed** (or handling seed that had already been treated)?

________________________________________________________

C17. Do you remember any years in which you did **<u>not</u>** use these **seed treatments**?

________________________________________________________

C18. In the course of a typical year, how many days did you use these **seed treatments**?

________________________ days

C18a. For how many hours each day? ___________________________ hours

C19. When using these **seed treatment**, did you use any equipment or clothing to stop you from breathing them in or to stop them from getting on your skin? ....... ☐ Yes
☐ No

C19a. **If yes**, what did you use(*be specific – eg latex/cloth/rubber gloves)*?

________________________________________________________

________________________________________________________

C20. Did you use **seed treatments** in any other way around your yard (eg lawn) or garden? ........................................................................ ☐ Yes
☐ No

C20a. What did you use? ____________________________________________

C20b. For how many days per year? ______________________________________

C21. I will now go over the seed treatment list previously sent to you. Please listen to this list of **seed treatments** used in Alberta and identify what you use/used and when you used them. **Indicate first year used, last year used, and the number of years used in total.**

| Seed Treatment | Calendar Year(s) | Seed Treatment | Calendar Year(s) | Seed Treatment | Calendar Year(s) |
|---|---|---|---|---|---|
| Benlate | F:<br>L:<br>T: | N-M Dual | F:<br>L:<br>T: | Tilt 250EC | F:<br>L:<br>T: |
| Benlate Toss-N-Go | F:<br>L:<br>T: | N-M Drillbox | F:<br>L:<br>T: | Tuberseal | F:<br>L:<br>T: |
| Benolin-R | F:<br>L:<br>T: | Polyram | F:<br>L:<br>T: | Vitaflo 280 | F:<br>L:<br>T: |
| DB Green | F:<br>L:<br>T: | Premiere Plus Flowable | F:<br>L:<br>T: | Vitavax Dual | F:<br>L:<br>T: |
| Dithane DG | F:<br>L:<br>T: | Ridomil/Bravo Twin Pack | F:<br>L:<br>T: | Vitavax Powder | F:<br>L:<br>T: |
| Foundation Seed Treatment | F:<br>L:<br>T: | Ridomil MZ 72WP | F:<br>L:<br>T: | Vitavax Single | F:<br>L:<br>T: |
| Lindane | F:<br>L:<br>T: | Ronilan | F:<br>L:<br>T: | Vitavax RS Flowable | F:<br>L:<br>T: |
| Manzate 200DF | F:<br>L:<br>T: | Rovral Flo | F:<br>L:<br>T: | | |
| Mertect | F:<br>L:<br>T: | Thiram 75WP | F:<br>L:<br>T: | | |

Others (please list any seed treatments used not listed above)

| Seed Treatment | Calendar Year(s) | Seed Treatment | Calendar Year(s) | Seed Treatment | Calendar Year(s) |
|---|---|---|---|---|---|
| | | | | | |
| | | | | | |
| | | | | | |

**Section D – Medical History and Current Health**

D1. Has a doctor ever told you that you had/have any of the following medical problems:

| Problem | Yes | No | Year and Details |
|---|---|---|---|
| Diabetes | ☐ | ☐ | |
| Cancer | ☐ | ☐ | |
| Seizures | ☐ | ☐ | |
| Head injury/concussion | ☐ | ☐ | |
| Thyroid trouble | ☐ | ☐ | |
| Stroke | ☐ | ☐ | |
| Brain tumor | ☐ | ☐ | |
| Kidney disease | ☐ | ☐ | |
| Allergies | ☐ | ☐ | |
| Arthritis | ☐ | ☐ | |
| Anaemia | ☐ | ☐ | |
| Heart problem | ☐ | ☐ | |
| High blood pressure | ☐ | ☐ | |
| Lead poisoning | ☐ | ☐ | |
| Epilepsy | ☐ | ☐ | |
| Jaundice (hepatitis) | ☐ | ☐ | |
| Other liver disease | ☐ | ☐ | |
| Tuberculosis | ☐ | ☐ | |
| Other lung disease | ☐ | ☐ | |
| Glandular fever | ☐ | ☐ | |
| Psychiatric illness | ☐ | ☐ | |
| Multiple sclerosis | ☐ | ☐ | |
| ME/Chronic fatigue syndrome | ☐ | ☐ | |
| Parkinson’s disease | ☐ | ☐ | |
| Fractures | ☐ | ☐ | |
| Osteoporosis | ☐ | ☐ | |
| Clots (deep vein thrombosis) | ☐ | ☐ | |
| Guillain-Barre Syndrome | ☐ | ☐ | |
| Myasthenia gravis | ☐ | ☐ | |

D2. Does you chest ever sound wheezy or whistling:

D2a. **when you have a cold**? .................................................. ☐ Yes ☐ No

D2b. **occasionally apart from colds**? .......................................... ☐ Yes ☐ No

D2c. **most days or nights**? ..................................................... ☐ Yes ☐ No

D3. **If D2a, D2b, OR D2c yes,** for how many years has this been present? _______________ (Number of years)

****Ask all****

D4. Have you ever had an attack of wheezing that has made you feel short of breath? ........................................................................ ☐ Yes ☐ No

D5. Has a doctor ever told you that you have asthma? ........................... ☐ Yes ☐ No

D5a. **If yes,** how old were you when this was first diagnosed? _____________

D6. In the past month, have you been bothered at all by any of the following:

| Problem | Not at all | Some what | Severely |
|---|---|---|---|
| Difficulty concentrating | ☐ | ☐ | ☐ |
| Headaches | ☐ | ☐ | ☐ |
| Feeling unhappy and depressed | ☐ | ☐ | ☐ |
| Waking up tied and worn out | ☐ | ☐ | ☐ |
| Too little energy to do things | ☐ | ☐ | ☐ |
| A poor memory | ☐ | ☐ | ☐ |
| Having to go back and check you have done things | ☐ | ☐ | ☐ |
| Difficulty in grasping what you read | ☐ | ☐ | ☐ |
| Cramps or spasms in your muscles | ☐ | ☐ | ☐ |
| The smell of paint, petrol or other chemicals | ☐ | ☐ | ☐ |
| Shortness of breath when walking with people of your own age | ☐ | ☐ | ☐ |
| Sudden changes of mood | ☐ | ☐ | ☐ |

## Section E – Recent exposure to pesticides

E1. Have you used / been exposed to **insecticides** in the past month?......... ☐ Yes ☐ No

E1a. **If yes**, what have you used? ____________________

____________________

E2. Do you think that any of the symptoms that you have experienced in the last month were due to this recent use of **insecticides**?........................................ ☐ Yes ☐ No

E2a. **If yes,** which symptoms?

____________________

____________________

E3. Have you used / been exposed to **herbicides** in the past month? .......... ☐ Yes ☐ No

E3a. **If yes**, what have you used? ____________________

____________________

E4. Do you think that any of the symptoms that you have experienced in the last month were due to this recent use of **herbicides**?.......................................... ☐ Yes ☐ No

E4a. **If yes,** which symptoms?

____________________

____________________

E5. Have you used / been exposed to **seed treatments** in the past month?... ☐ Yes ☐ No

E5a. **If yes**, what have you used? ____________________

____________________

E6. Do you think that any of the symptoms that you have experienced in the last month were due to this recent use of **seed treatments**? .................................. ☐ Yes ☐ No

E6a. **If yes,** which symptoms?

____________________

____________________

## Section F – Future contact

F1. When we have completed the current study, we will be writing a report. Would you like us to send a summary of the findings?........................................... ☐ Yes
☐ No

F2. When we have these results, we may want to visit some of the farmers to carry out some simple health tests and to ask a few more questions.

Could we contact you again later if you are one of those chosen for this further study? ............................................................................ ☐ Yes
☐ No

F3. Finally, is there anything else you would like to tells us about the use of pesticides and the way you think it can affect people's health?

______________________________________________

______________________________________________

______________________________________________

______________________________________________

______________________________________________

______________________________________________

______________________________________________

______________________________________________

______________________________________________

______________________________________________

****Thank you very much for taking part in this study. We are most grateful for your help.****
